# Genetic identification and expression optimization of a novel protease HapR from *Bacillus velezensis*

**DOI:** 10.3389/fbioe.2024.1383083

**Published:** 2024-03-13

**Authors:** Zhenying Han, Changwen Ye, Xinyu Dong, Chenchen Chen, Dian Zou, Kuo Huang, Xuetuan Wei

**Affiliations:** ^1^ State Key Laboratory of Agricultural Microbiology, College of Food Science and Technology, Huazhong Agricultural University, Wuhan, China; ^2^ Zhengzhou Tobacco Research Institute of China National Tobacco Corporation, Zhengzhou, China; ^3^ Shenzhen Institute of Nutrition and Health, Huazhong Agricultural University, Wuhan, China; ^4^ Shenzhen Branch, Guangdong Laboratory for Lingnan Modern Agriculture, Genome Analysis Laboratory of the Ministry of Agriculture, Agricultural Genomics Institute at Shenzhen, Chinese Academy of Agricultural Sciences, Shenzhen, China

**Keywords:** protease, *Bacillus* velezensis, whole genome sequencing, heterologous expression, expression optimization

## Abstract

Due to the broad application and substantial market demand for proteases, it was vital to explore the novel and efficient protease resources. The aim of this study was to identify the novel protease for tobacco protein degradation and optimize the expression levels. Firstly, the tobacco protein was used as the sole nitrogen resource for isolation of protease-producing strains, and a strain with high protease production ability was obtained, identified as *Bacillus velezensis* WH-7. Then, the whole genome sequencing was conducted on the strain *B. velezensis* WH-7, and 7 proteases genes were mined by gene annotation analysis. By further heterologous expression of the 7 protease genes, the key protease HapR was identified with the highest protease activity (144.19 U/mL). Moreover, the catalysis mechanism of HapR was explained by amino acid sequence analysis. The expression levels of protease HapR were further improved through optimization of promoter, signal peptide and host strain, and the maximum protease activity reaced 384.27 U/mL in WX-02/pHY-P43-SP_yfkD_-*hapR*, increased by 167% than that of initial recombinant strain HZ/pHY-P43-SP_hapR_-*hapR*. This study identified a novel protease HapR and the expression level was significantly improved, which provided an important enzyme resource for the development of enzyme preparations in tobacco protein degradation.

## Graphical Abstract

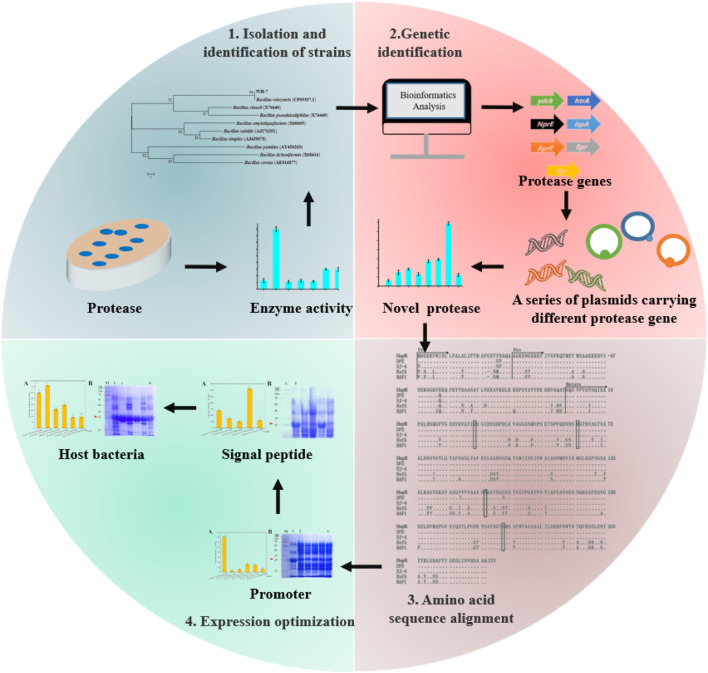

## 1 Introduction

Improvement of the tobacco quality is important for reduction of health hazards for smokers (Pauly and Paszkiewicz, 2011; Wei et al., 2014; Flor et al., 2021). The contents of biomacromolecules such as proteins and polysaccharides significantly affect the tobacco quality. Excessive protein content in tobacco is more harmful for the human health, and it can lead to the generation of noxious odors during tobacco combustion, including polycyclic aromatic hydrocarbons (Lee et al., 2011), free radicals (Di Meo and Venditti, 2020), and nitrosamines (Beard and Swager, 2021). Moreover, excessive protein can reduce the burning rate and produce burnt feathers smell, seriously threatening the sensory quality of tobacco (Wen et al., 2023). Consequently, control of the protein level in tobacco is crucial for the reduction of health hazards and improvement the tobacco quality.

Protease can catalyze the hydrolysis of peptide bonds, and it is one of the most important industrial enzymes (Contesini et al., 2018). Various proteases have been broadly applied in the food, feed, pharmaceutical, laundry, and other industries (dos Santos Aguilar and Sato, 2018; Sabotič and Kos, 2012; Tavano et al., 2018). In recent years, several reports demonstrated that application of proteases could reduce protein content and ultimately enhance the overall quality of tobacco product. In a previous study, the protein content of tobacco was reduced by 59% after adding protease, and the content of nitrogenous toxins (e.g., 4-aminobiphenyls and hydrogen cyanide produced by combustion of amino acids and proteins) was also significantly decreased, which improved the quality and safety of tobacco (Liu et al., 2011). Currently, the proteases used for tobacco processing were predominantly from commercially available food-grade proteases (Contesini et al., 2018). However, these traditional proteases applied for food had certain limitations. Due to substantial disparities between tobacco and common food matrices, food-grade proteases exhibited low activity and limited specificity in degrading proteins of tobacco (Li et al., 2013).

Proteases can be isolated from plants, animals, and microorganisms. Microorganisms serve as an important sources of proteases, and a number of wild-type bacteria have been isolated for the production of proteases. However, the natural yields of proteases from wild bacteria are relatively low. For example, Ma et al. isolated a strain of *B.acillus subtilis* XP01 which could hydrolyzed both starch and protein in tobacco, while the protease activity of this wild strain was only 14.29 U/mL (Ma et al., 2023). By comparing the different sizes of protein hydrolysis zones, Ning et al. isolated a protease-producing strain of *B. subtilis* B1 from tobacco, which could promote the degradation of tobacco proteins and improve the microbial community structure in tobacco. However, the protease activity of *B. subtilis* B1 was just 10.75 U/mL (Ning et al., 2023). Consequently, exploring more efficient tobacco protein-degrading strain with high protease activity by natural isolation or genetic engineering is crucial.

In order to obtain the efficient enzyme-producing strain, various genetic engineering strategies have been used for optimization of enzyme expression, such as promoter engineering, signal peptide screening and host strain selection (Degering et al., 2010; Zhang et al., 2017; Contesini et al., 2018). Wu et al. increased β-glucuronidase (GUS) expression by 249% through promoter engineering (Wu et al., 2011). Caspers et al. showed that optimization of signal peptide resulted in an 400% increase of keratinase activity (Caspers et al., 2010). However, effects of promoters and signal peptides on different enzymes are usually different, and the optimal promoter or signal peptide is not suitable for all heterologous enzymes. In order to obtain the efficient protease-producing strain, tobacco leaves and soil samples from 8 representative ecological zones in China were collected to screen the target strains using tobacco protein as the sole nitrogen. One efficient strain was isolated and identified as *B. velezensis* WH-7, which had a significant capacity for degrading tobacco proteins. Subsequently, whole genome sequencing and annotation of *B. velezensis* WH-7 were carried out, and 7 protease genes were expressed and compared in *Bacillus amyloliquefaciens* HZ-12 to identify the key protease. Moreover, the expression level of protease was further enhanced by optimizing the promoters, signal peptides and host strains. This study not only excavates a key protease and its gene resource, but also provides an efficient engineering strain for protease production, which will promote the development of new enzyme preparations for degradation of tobacco protein.

## 2 Materials and methods

### 2.1 Samples and chemicals

The representative tobacco rhizosphere leaves and soil samples were collected from China as listed in Supplementary Table S1. The samples were collected using the five-points sampling method. Each sample point contained 200 g tobacco leaves and soil, and a total of three parallel samples were mixed evenly. All chemicals were purchased from Sinopharm Chemical Reagent Co., Ltd. (Shanghai, China).

### 2.2 Isolation and identification of the bacterium

Tobacco samples were taken 10 g, mixed with 100 mL sterile water, and treated at 180 rpm for 1 h. The supernatant was heated in a water bath at 80°C for 15 min to retain heat-resistant strains, and 0.5 mL supernatant was added to 25 mL of enrichment medium using tobacco protein substrate as the sole nitrogen source (2 g/L tobacco protein), cultured at 37°C in 180 rpm for 24 h. The fermentation broth was diluted to 10^−4^, 10^−5^, 10^−6^, and spread onto milk powder plates (10 g/L peptone, 10 g/L NaCl, 5 g/L yeast extract, 10 g/L skim milk powder). After incubation at 37°C for 24 h, the colonies surrounded by broad transparent zones were inoculated into LB liquid medium (10 g/L peptone, 5 g/L yeast extract, 10 g/L NaCl), cultured for 24 h at 37°C in 180 rpm. After centrifugation at 9,500 g for 10 min, the supernatant was spotted onto milk powder plates at 37°C for 24 h, and then the diameter of the transparent zone was observed and measured.

The optimal strain was identified by 16S rRNA gene sequence analysis. The 16S rRNA gene was amplified using primers (27f, AGA​GTT​TGA​TCC​TGG​CTC​AG) and (1492r, GGT​TAC​CTT​GTT​ACG​ACT​T) (Thomas, 2004). The 16S rRNA gene fragment was sequenced by Tsingke Biotech Co., Ltd. The sequence was compared to the GenBank DNA database by the Blast program (http://blast.ncbi.nlm.nih.gov/Blast.cgi), and a phylogenetic tree was constructed with MEGA 7.0 software.

### 2.3 Bacterial culture and genomic DNA extraction

Since the optimal strain WH-7 was identified as *B. velezensis*, its total DNA was extracted for genome sequencing. One single colony of *B. velezensis* WH-7 was inoculated into 50 mL of LB liquid medium in 250 mL flasks, which were incubated at 37°C in 180 rpm for 8 h to generate the seed culture. Then, 1.5 mL seed culture were transferred into 50 mL fermentation broth in 250 mL flasks, incubated at 37°C, 180 rpm for 8 h. The broth was subsequently centrifuged with 1,520 g at 4°C for 10 min, and the bacterial cells were collected. Then, the total DNA of *B. velezensis* WH-7 was extracted using a bacterial genomic DNA isolation kit (Vazyme, Nanjing, China), and sent to Novogene Co., Ltd. for sequencing analysis (Lai et al., 2023).

### 2.4 Whole-genome sequencing and analysis

DNA samples were randomly broken into fragments with approximately 350 bp in length by a Covaris ultrasonic fragmentation machine. The DNA libraries were constructed by the NEBNext®Ultra™ DNA Library Prep Kitfor Illumina (NEB, United States) kit. Then, the libraries were initially quantified with Qubit 2.0, followed by the detection of the insert fragments by using Agilent 2,100. When the insert size met the expectation, the concentrations of the libraries were accurately quantified using q-PCR method to ensure the quality of the libraries. Then, the libraries were sequenced by Illumina NovaSeq PE150 according to the effective concentration and the target downstream data volume. The Canu software (https://github.com/marbl/canu/, version: 2.0) was used to perform genome assembly of the reads, and preliminary assembly results were obtained. Then, Racon (version: 1.4.13) software was used to perform three rounds of error correction on the spliced results based on three generations of sequencing data. The Pilon software (version: 1.22) of second-generation reads was used for three rounds of error correction to obtain the final assembly results. The functional annotation of the genome was performed, and the protein sequences of the predicted genes were compared with COG and KEGG functional databases for Diamond comparison. For each sequence, the comparison result with the highest score (default identity ≥40% and coverage ≥40%) was selected for annotation (Avsec et al., 2021).

### 2.5 Analysis and prediction of protease in *B. velezensis* WH-7

To predict the proteases in *B. velezensis* strain WH-7, we employed COG and KEGG databases. Furthermore, the SignalP 5.0 software was utilized to predict protein signal peptides and identify specific signal peptide. In addition, TMHMM v2.0 was used to predict transmembrane domains of the protease. Moreover, the sequences of the targeted proteases from *B. velezensis* WH-7 were compared with gene sequences available in the NCBI database.

### 2.6 Recombinant expression of the protease genes

To screen the key protease genes, the predicted genes were expressed in *B. amyloliquefaciens* HZ-12 following our previous methods (Zou et al., 2020; Chen et al., 2022; Jiang et al., 2022). For instance, the *hapR* gene from *B. velezensis* WH-7, the promoter P43 from *B. subtilis* 168, and the TamyL terminator from *Bacillus licheniformis* were amplified using corresponding primers. Subsequently, the three fragments P43, *hapR* and TamyL were fused through SOE-PCR. This fused fragment was then inserted into the pHY300PLK plasmid between the *Sma*I and *Xba*I restriction sites. Positive clones were identified by colony PCR and sequencing analysis. Finally, the expression plasmid pHY-*hapR* was introduced into HZ-12 by electro-transformation, generating the desired recombinant strain. Other recombinant strains were also constructed using the same method in this study. Similarly, the exogenous signal peptide sequence (Supplementary Table S4) was fused with the gene by the SOE-PCR (Zhao et al., 2024). Supplementary Table S2 provided the strains and plasmids utilized in this study, and all designed primers were listed in Supplementary Table S3.

### 2.7 SDS-PAGE analysis of protease expression

Protein samples were prepared according to our previous methods (Jiang et al., 2022). Subsequently, the samples were analyzed by electrophoresis on 12% SDS-PAGE polyacrylamide gels. The gel was color stained with Coomassie Brilliant Blue R-250 in methanol-acetic acid-water (4.5:1:4.5, v/v) and decolorized in methanol-acetic acid-water (2.5:0.8:6.7, v/v).

### 2.8 Detection of protease activity

The diluted enzyme solution (1 mL) was added to tube A (as the blank control group) and tube B (for activity measurement). After incubation at 40°C for 2 min, the tube A was supplemented with 2 mL trichloroacetic acid (64.70 g/L), while the tube B was added with 1 mL casein solution (10 g/L). Both tubes were further heated at 40°C for 10 min. Then, 1 mL casein solution was added to tube A, and 2 mL trichloroacetic acid was transferred to tube B. The mixtures were left at 25°C for 20 min, filtered to obtain the supernatant. Additionally, 5 mL sodium carbonate solution and 1 mL folinic acid phenol reagent was added into the supernatant. Subsequently, the mixtures were thoroughly mixed and heated at 40°C for 20 min. The absorbance of the mixture was measured at a wavelength of 680 nm. The enzyme solution dilution was determined based on the L-Tyrosine standard curve. The enzymatic activity of the samples was calculated using the following formula, in which A and n indicate enzyme activity of the final dilution derived from the standard curve and dilution factor, respectively:

$$\text{Enzyme activity}\left(U/\text{mL}\right)=\frac{A\times 4\times N}{10}$$

### 2.9 Statistical analysis

Each fermentation experiment was carried out at least in triplicate. SPSS 25.0 was used for statistical analysis, calculating the means and standard deviations, and evaluating the significance. Origin 8.5 was used to deal with the data and draw the graphs.

## 3 Results and discussion

### 3.1 Screening and identification of high-yield protease strains

Studies have shown that tobacco was rich in soluble plant proteins (Vansuyt et al., 2003). Microorganisms originated from the tobacco environment might easily exhibited significant ability to hydrolyze tobacco proteins. Using tobacco protein substrate as the sole nitrogen source was beneficial for isolation of protease-producing strains. Therefore, the samples of tobacco leaves and rhizosphere soil were selected from 8 representative tobacco cultivation regions of China, and the enrichment medium with tobacco protein as the sole nitrogen source was used for isolation the target strain. After enrichment culture and transparent circle observation, 7 strains with relatively large transparent circles were isolated, and their protease fermentation activities were further analyzed and compared.

**FIGURE 1:**
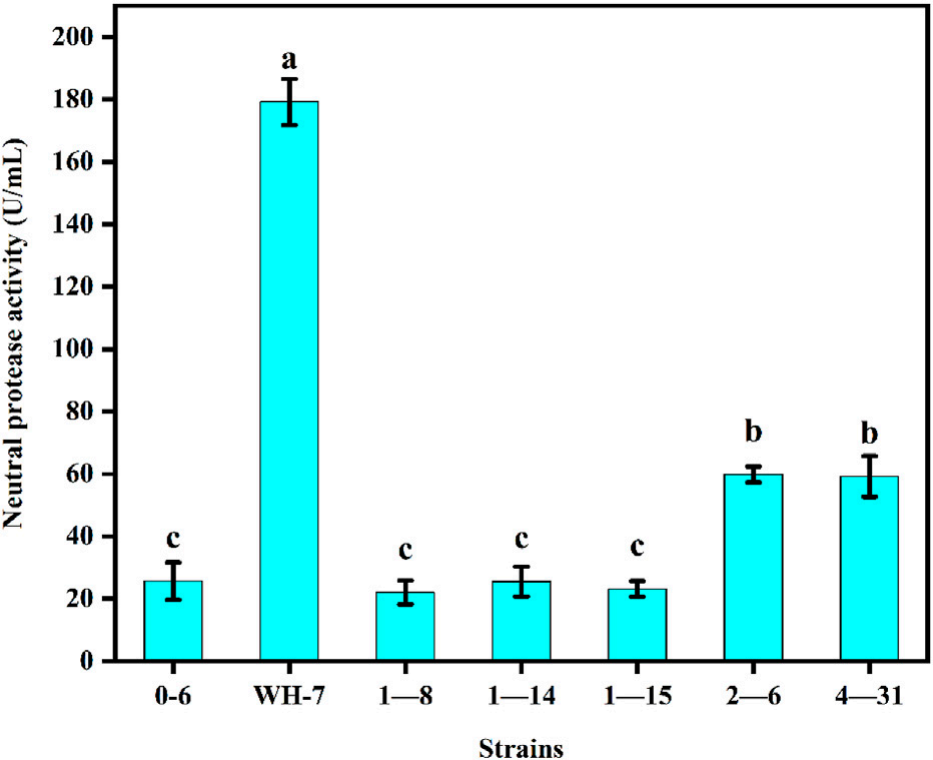
Protease production abilities of 7 strains. Values are expressed as averages ±SD. Different letters indicate significant differences (*p* < 0.05) among groups.

**FIGURE 2:**
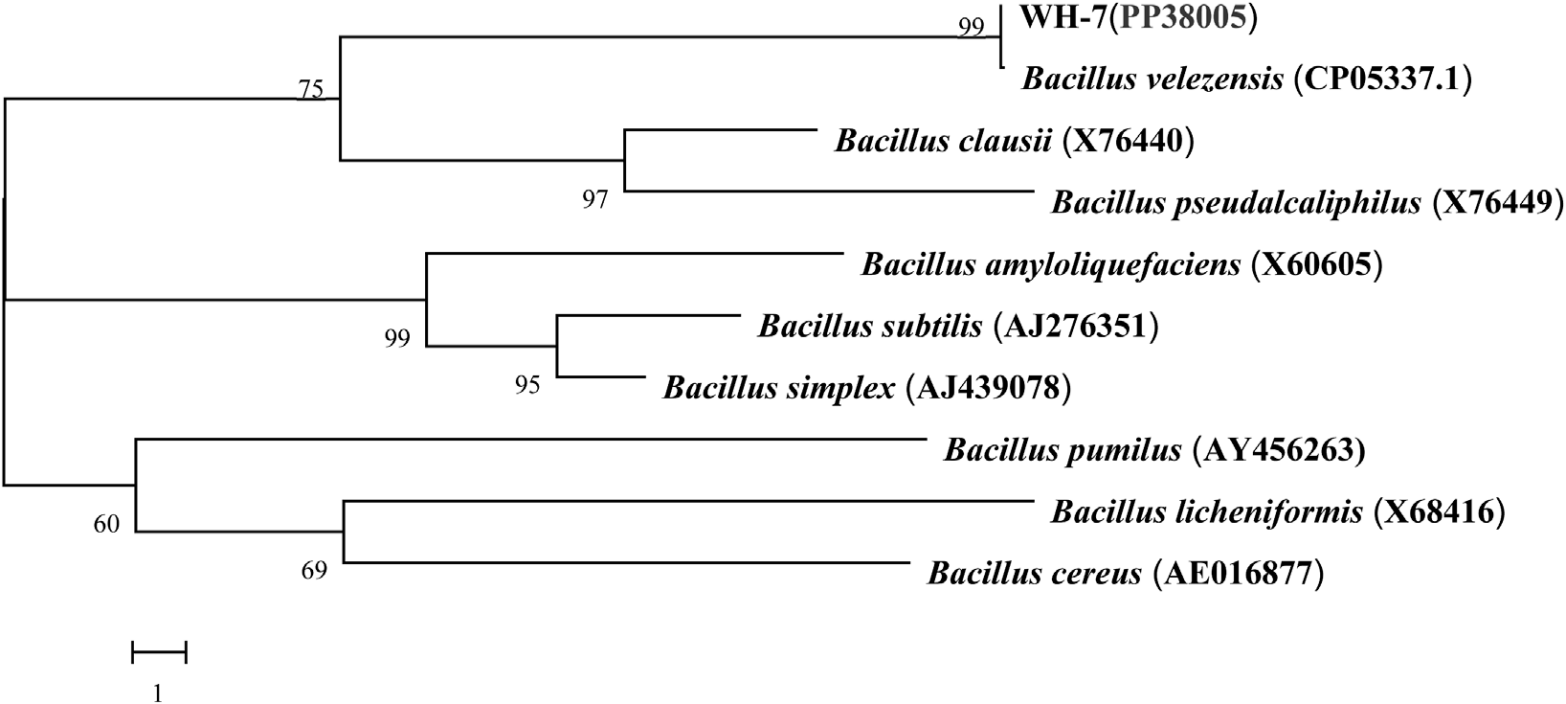
Phylogenetic tree of WH-7 based on 16S rRNA gene sequences. Numbers in parentheses indicates the sequence accession numbers of the representative organisms. The scale bar represents 1 nucleotide substitution per position.

As shown in Figure 1, the protease fermentation activities of these 7 strains were in the range of 23.56–186.32 U/mL, and the WH-7 strain exhibited the highest protease activity compared to other strains. Then, the strain WH-7 was identified through 16S rRNA gene sequence analysis. Compared with sequences in the GenBank database, the 16S rRNA gene sequence of WH-7 showed 99% similarity with that of the *B. velezensis* (CP05337.1). In order to further verify the genetic relationship of the strain WH-7, 9 representative *Bacillus* strains were selected to construct a phylogenetic tree. As shown in Figure 2, WH-7 had the highest affinity with *B. velezensis*, further confirming that the strain WH-7 belonged to *B. velezensis*. The 16S rRNA gene sequence accession number is PP380005. According to previous studies, most of the commercial proteases were from the *Bacillus* sp. such as *Bacillus amyloliquefaciens*, *Bacillus subtilis* and *Bacillus licheniformis*. These *Bacillus* sp. had been widely investigated, and applied to produce large amount of neutral and alkaline proteases (Contesini et al., 2018). Recently, *Bacillus velezensis* had also been preliminarily investigated to produce protease (Khalid et al., 2021). However, the overall profile of protease-producing *Bacillus velezensis* and the key protease genes had not been deeply investigated.

### 3.2 Analysis of the whole genome of strain WH-7

**FIGURE 3:**
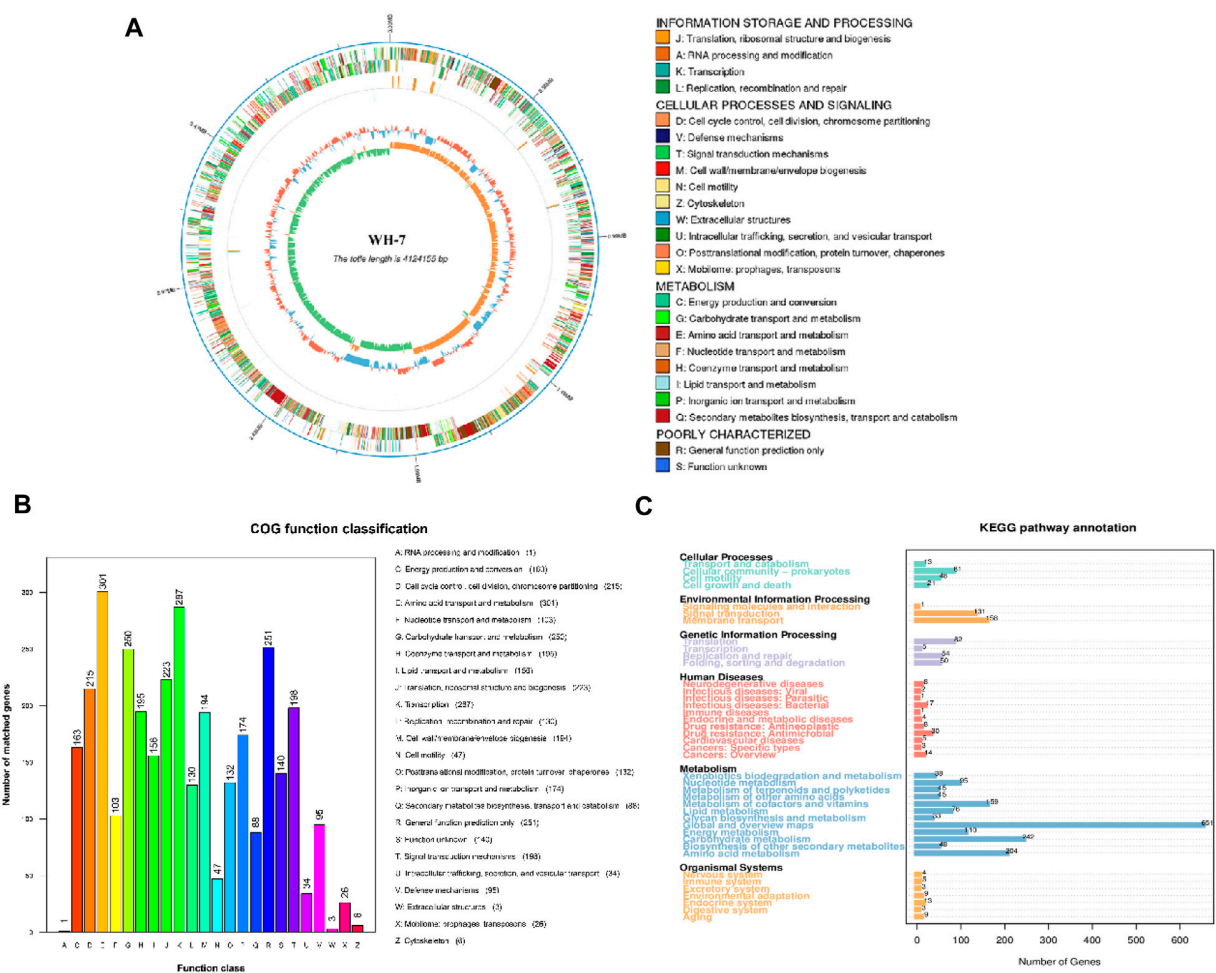
Genome analysis of WH-7. **(A)** Circular genome map of WH-7. Note: The outermost circle is the genomic sequence position coordinates, from outside to inside, the gene function annotation results (including COG annotation results information), ncRNA, genomic GC content: the GC content is counted by window (chromosome length/1,000) bp, step (chromosome length/1,000) bp, the inward blue part indicates that the GC content of the region is lower than the average GC content of the whole genome. The outward red part indicates that the GC content of the region is lower than the average GC content of the whole genome, and the higher the peak value indicates that the difference with the average GC content is larger. Genomic GC skew value: window (chromosome length/1,000) bp, step (chromosome length/1,000) bp, the specific algorithm is G-C/G + C, the inward green part indicates that the G content of the region is lower than the C content, and the outward orange part is the opposite. **(B)** Strain WH-7 general protein prediction COG database. Gene function annotation COG function classification chart: horizontal coordinates indicate COG function type, vertical coordinates indicate the number of genes on the annotation. **(C)** Strain WH-7 general protein prediction KEGG database. Gene function annotation KEGG metabolic pathway classification chart: the number on the bar represents the number of genes on the annotation; the remaining axis is the code of each functional class in the database.

In this study, we had demonstrated that the strain of *B. velezensis* WH-7 could hydrolyzed the tobacco protein. To deeply explore the key protease for protease-producing from *B. velezensis* WH-7, whole genome sequencing and analysis were performed on *B. velezensis* WH-7. The whole genome data accession number is CP145817. As shown in Table 1, the genome of *B. velezensis* WH-7 had a total length of 41,241,55 bp including 4,386 genes, and these genes accounted for 89.94% of the total genome length. Additionally, we also identified 198 tandem repeats, spanning a total length of 15,184 bp, and it accounted for 0.37% of the entire genome length. Subsequently, the assembled genome sequence of *B*. *velezensis* WH-7 was visually represented (Figure 3A), integrated the predicted results of coding genes and proceeded gene functional annotation analysis. Regarding these genomic features including gene size and GC content, strain WH-7 was similar to those of *B. velezensis* DMB06 and *B. velezensis* KMU01, and none of them contain plasmids (Heo et al., 2021; Na et al., 2022).

**TABLE 1:** Genome statistics of the *B. velezensis* WH-7.

Features	Value
Total Length (bp)	4,124,155
GC (%)	46.15
Gene Number	4,386
Total Genes’ Length (bp)	3,709,230
Gene Avrage Length (bp)	849
Gene Length/Genome (%)	89.94
Tandem Repeat Number	196
Tandem Repeat Length (bp)	9–282
Minisatellite DNA Number	157
Microsatellite DNA Number	0
rRNA Number	27
tRNA Number	86
sRNA Number	0
Genomics Islandsnumber	7
Prophagenumber	11

In order to future analyze gene function accurately, this study annotated the genome of *B*. *velezensis* WH-7 strain by comparing with COG and KEGG databases. According to the latest reported COG database, a total of 3,036 genes were successfully annotated (Figure 3B). Therein, 301 genes were associated with amino acid transport and metabolism, 103 genes were involved in nucleotide transport and metabolism, as well as 195 genes were related to coenzyme transport and metabolism. These results showed that the number of genes related to energy metabolism and defense mechanisms were enriched in *B. velezensis* WH-7. However, the strain-specific and accessory genes could not be categorized using the COG database, and relationships between key metabolic genes of the strain were not entirely predicted. Since the KEGG database could systematically analyze the metabolic pathways and functions of gene products and compounds (Okuda et al., 2008), we conducted BLAST analysis of the amino acid sequence of WH-7 with the KEGG database. As shown in Figure 3C, a total of 4,120 genes were annotated in strain WH-7. Meanwhile, the analysis showed that the genes related to metabolism in strain WH-7 accounted for 69% of the total number of genes, of which the energy metabolism genes accounted for about 6%. The results were consistent with the COG analysis. The results predicted that 110 genes were associated with energy metabolism (Figure 3C), which suggested that strain WH-7 had a high metabolic energy (Reuss, 2008). Combining the results of above four databases, the energy metabolism was the dominant component in WH-7. Previous studies also showed that *B. amyloliquefaciens* had efficient energy metabolism due to the distribution of energy metabolism related genes (Chun et al., 2019). Notably, Fischer et al. suggested that the protease played the central role in energy metabolism (Fischer et al., 2015). This might be the main reason that WH-7 with a high protease activity had efficient energy metabolism.

### 3.3 Screening of protease genes in the *B. velezensis* WH-7 strain

The protease gene sequences of *B*. *velezensis* WH-7 were predicted by comparing with COG and KEGG database, and their location in the cell (extracellular, intracellular and cell membrane) were also predicted. Moreover, the predicted proteases were corrected using the reported proteome of *B*. *velezensis*, and their protein molecular weights were calculated by Edit Seq. In total, 103 known and putative proteases were obtained from *B*. *velezensis* WH-7 including 13 extracellular proteases, 49 intracellular proteases, and 41 proteases bound to cell membrane or cell wall.

According to previous reports, proteases were mainly divided into four categories, including serine proteases, metalloproteases, aspartic proteases, and cysteine proteases (Kraut, 1977). Among them, serine proteases mainly secreted by *Bacillus* sp. had been extensively studied (Li et al., 2022), and the active center was triple catalyst Asp-His-Ser (Almog et al., 2002). Metalloproteinases existed in fungi, bacteria and actinomycetes, and the active center was divalent metal ions, such as Zn^2+^ and Ca^2+^ (Raeeszadeh-Sarmazdeh et al., 2020). In addition, the active center of aspartic protease was aspartic acid (Feliciano and Gold, 2020). The major extracellular protease genes identified in the *Bacillus* are *aprE* (Stahl and Ferrari, 1984), *nprE* (Yang et al., 1984), *vpr* (Lainšček et al., 2018), and *epr* (Lanigan-Gerdes et al., 2007). The protease HtrA had been reported to have high sequential and structural homologies with the serine protease, and the IspA had also been reported to contain the triple catalyst Asp-His-Ser of serine proteases (Fujisaki et al., 1990). Ydck was predicted to be a serine protease in whole genome sequencing analysis, which had not been confirmed. In WH-7, similar genes were also found in the genome (Table 2) including two potential metalloproteases (named *ydcK*, *nprE*) and five serine proteases (named *hapR*, *ispA*, *vpr*, *epr, htrA*). Lai et al. isolated a protease-producing strain *Bacillus* sp. CN2, and 4 aspartic proteases, 30 cysteine proteases, 55 metalloproteases, and 56 serine proteases were predicted (Lai et al., 2021). However, the aspartic protease and cysteine proteases were not found in WH-07 according to whole genome sequence.

**TABLE 2:** The main predicted proteases of strain WH-7.

Gene name	Description	Molecular weight (KDa)	Location
*hapR*	extracellular serine alkaline protease	39.07	Extracellular
*vpr*	extracellular serine protease	85.84	Extracellular
*epr*	extracellular serine protease	61.69	Extracellular
*nprE*	extracellular neutral metalloprotease	59.16	Cell wall
*htrA*	serine protease	47.22	Chloroplast
*ispA*	intracellular serine protease	35.35	Cell wall
*ydck*	hypothetical protease	19.76	Cytoplasmic

### 3.4 Heterologous expression of different protease genes

**FIGURE 4:**
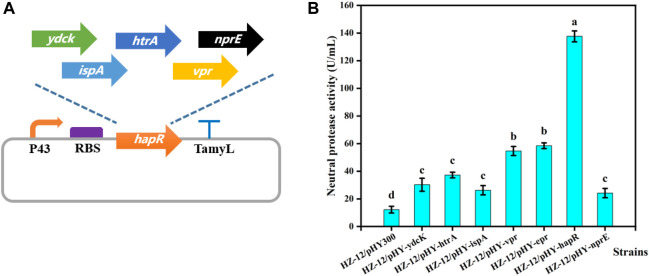
Heterologous expression of different proteases. Note: **(A)** Construction of different protease expression vectors. **(B)** Comparison of extracellular protease activity of recombinant strains with different protease genes.

**FIGURE 5:**
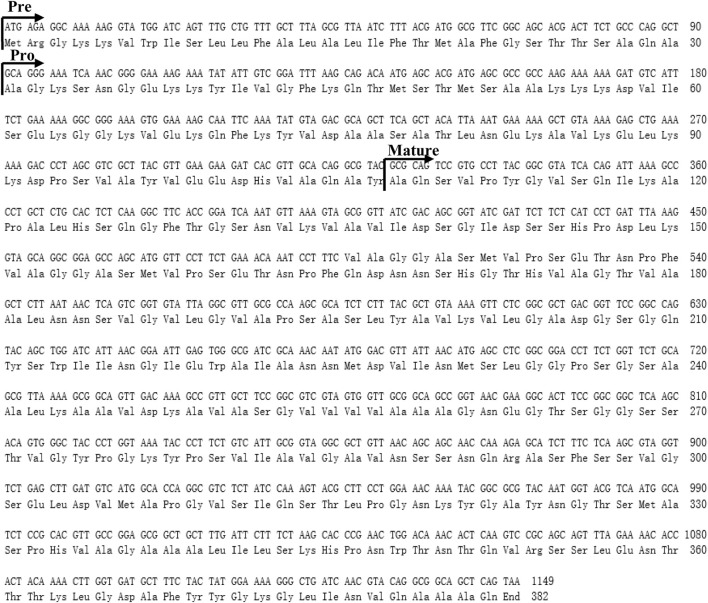
Nucleotide (upper line) and deduced amino acid (lower line) sequences of the protease HapR. The predicted signal peptide, pro-peptide, and mature peptide (mature) were marked with arrows.

To mine the key protease gene, as-predicted 7 proteases genes of *B. velezensis* WH-7 were expressed, and the recombinant strains were constructed using the previously reported method (Zou et al., 2020). *B. amyloliquefaciens* HZ-12 was used as the host strain, and pHY300PLK served as the basic expression vector (Figure 4A). A total of 7 recombinant strains were successfully obtained, namely, HZ/pHY-*ydcK*, HZ/pHY-*htrA*, HZ/pHY-*ispA*, HZ/pHY-*vpr*, HZ/pHY-*epr*, HZ/pHY-*hapR* and HZ/pHY-*nprE* respectively. Using HZ/pHY300 as the control strain, the fermentation activities of 8 recombinant strains were measured and compared. As shown in Figure 5B, the recombinant strain HZ/pHY-*hapR* had the highest protease activity (144.19 U/mL), which was much higher than that of other genes. This result suggested that the gene *hapR* of *B. velezensis* WH-7 was the key protease gene for degrading proteins in tobacco. Previous studies demonstrated that there are different proteases in *Bacillus* species, such as AprE (Hata et al., 2001; Sareen et al., 2005; Shi et al., 2019), subtilisin DJ-4 (AY627764) (Choi et al., 2004), DFE (DQ132806) (Peng et al., 2003), BsfA (JN392072.1) (Hu et al., 2019), BAF1 (FJ517584.1) (Agrebi et al., 2009). Herein, the protease HapR was found to be the key protease in *B. velezensis* for the first time.

### 3.5 Sequence analysis of the protease HapR and deduced amino acids

**FIGURE 6:**
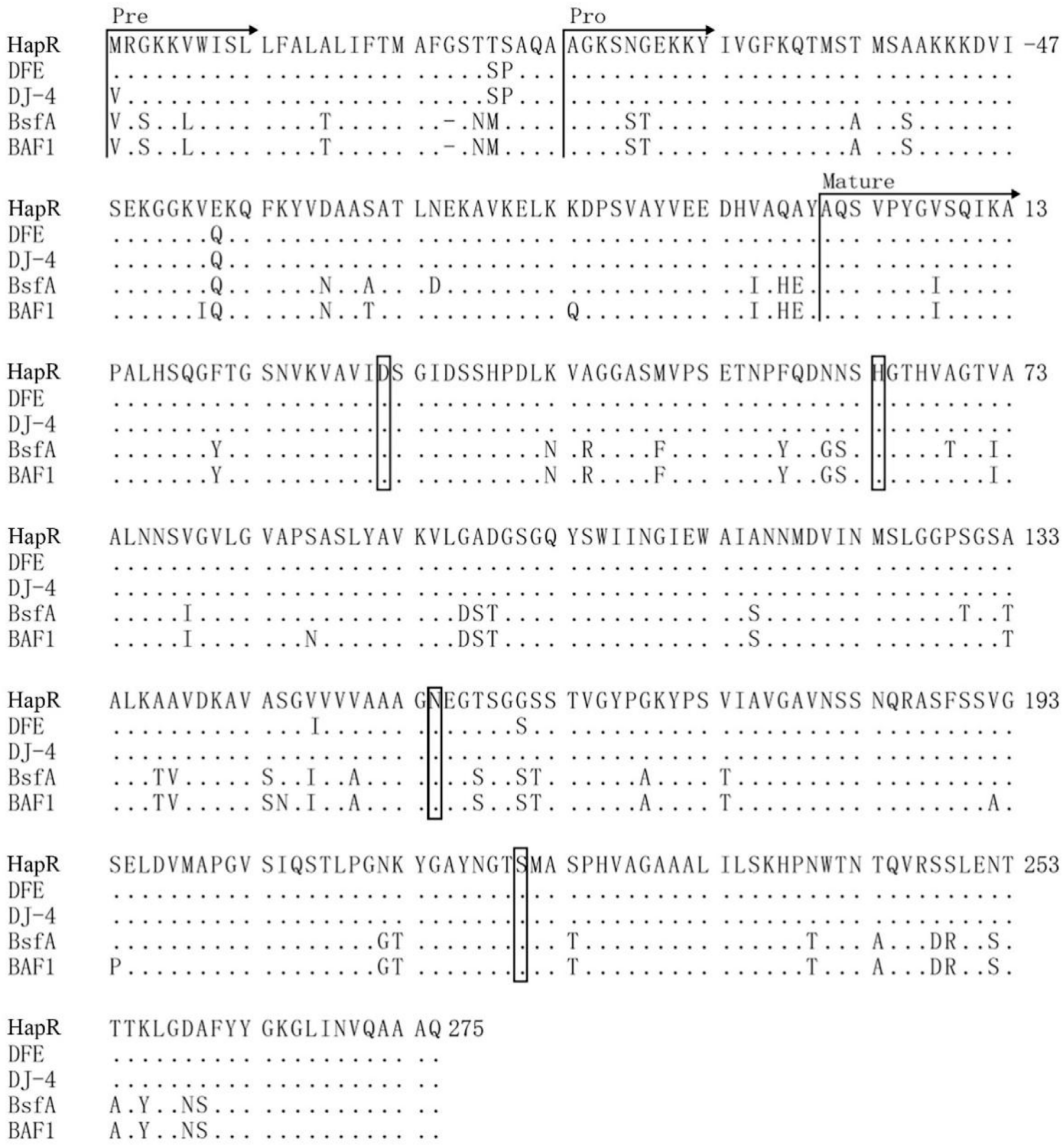
Amino acid sequence alignment of the protease HapR, with subtilisin (DJ-4, DFE) from (B) *amyloliquefaciens* and subtilisin (BAF1 BsfA) from (B) *subtilis*. The signal peptide, pro-peptide, and mature peptide (mature) were indicated with arrows. “.” indicated the same residue as the first sequence. The catalytic center residues (Asp-32, His-64, and Ser-221) were boxed. The carbonyl amide side chain of Asn-155 were boxed. The initial amino acid of the mature peptide was numbered as + 1.

Based on above results, this study found that the protease HapR of *B. velezensis* WH-7 played the most key role in degrading proteins in tobacco. To further comprehensively explain the function of the gene *hapR* of *B. velezensis* WH-7, the sequence of *hapR* gene was translated into 382 amino acids by BioEdit software. The amino acid sequence was further analyzed using SignalP 5.0. As shown in Figure 5, the result indicated that protease HapR of *B. velezensis* WH-7 consisted of a pre-sequence with 30 amino acids, a pre-peptide with 77 amino acids, and a mature-peptide with 275 amino acids respectively. Then, it was compared with the sequences of four known proteases, including subtilisin DJ-4 (AY627764) (Choi et al., 2004), DFE (DQ132806) (Peng et al., 2003), BsfA (JN392072.1) (Hu et al., 2019), BAF1 (FJ517584.1) (Agrebi et al., 2009). As shown in Figure 6, the amino acid sequence of HapR exhibited 98.95% homology with subtilisin DJ-4, 98.02% homology with subtilisin DFE, 85.13% homology with subtilisin BsfA, and 84.29% homology with subtilisin BAF1.

**FIGURE 7:**
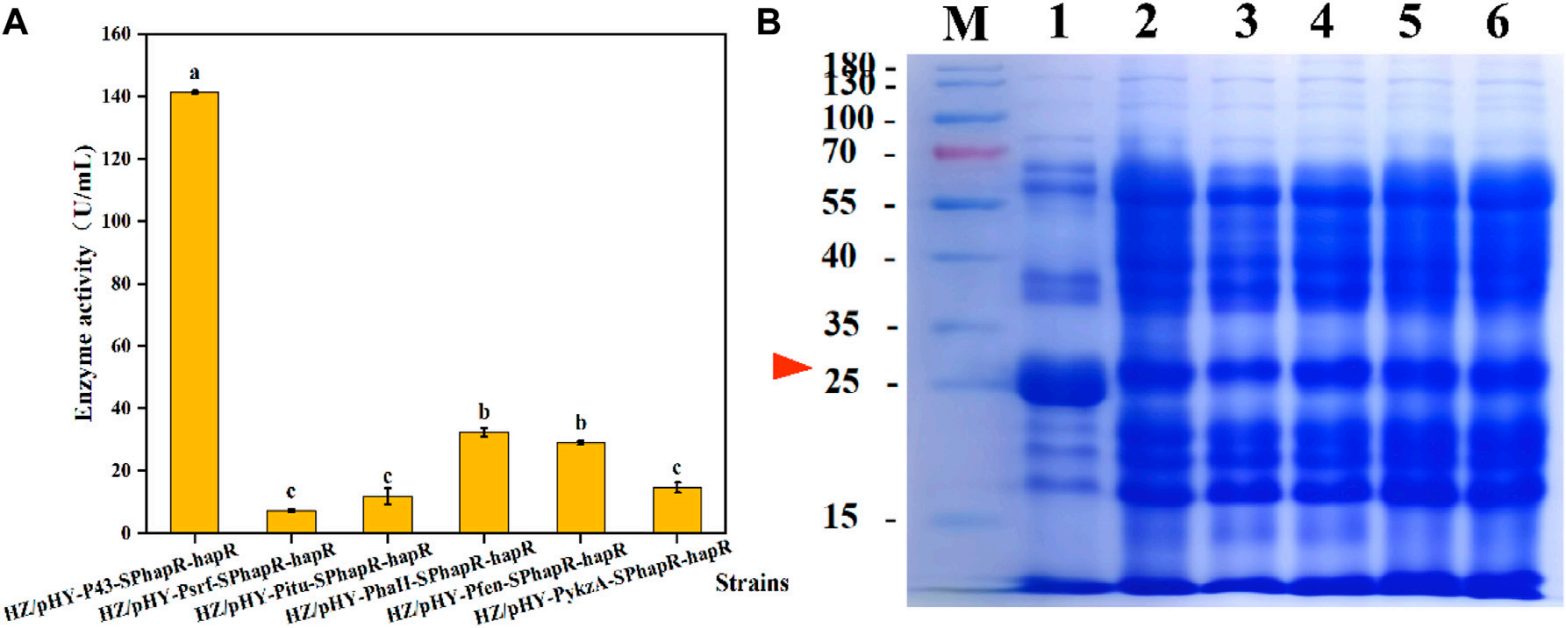
Effects of different promoters on expression levels of protease HapR. **(A)** Comparison of extracellular protease activities of recombinant strains with different promoters. **(B)** SDS-PAGE analysis of the fermentation supernatant of different strains. M: Marker; 1–6: HZ/pHY-P43-SP_hapR_-*hapR*, HZ/pHY-Psrf-SP_hapR_-*hapR*, HZ/pHY-Pitu-SP_hapR_-*hapR*, HZ/pHY-PhaII-SP_hapR_-*hapR*, HZ/pHY-Pfen-SP_hapR_-*hapR*, and HZ/pHY-PykzA-SP_hapR_-*hapR*.

For the degradation mechanism of serine proteases, Asp-32, His-64 and Ser-221 were considered to be the conserved catalytic center (Nakamura et al., 1992). As shown in Figure 7, HapR contained these three amino acid residues (Asp-32, His-64 and Ser-221). Among them, the carboxyl ester side chain of Asp-32 formed a hydrogen bond with the proton of the imidazole ring of His-64, and another nitrogen on His-64 formed a hydrogen bond with the OH proton of Ser-221, which caused the charge separation of hydroxyl occurs. Subsequently, the oxygen atom of Ser-221 proceeded to attack the incoming substrate with the help of the adjacent carbonyl amide side chain of Asn-155, achieved the degradation of macromolecular proteins (Carter and Wells, 1988).

### 3.6 Effects of different promoters on the expression levels of protease HapR

An appropriate promoter would facilitate efficient expression of recombinant proteins in *Bacillus* (Cheng et al., 2016). To further enhance the expression level of the protease HapR, 5 constitutive promoters were compared with the HZ/pHY-P43-SP_hapR_-*hapR* harboring the promoter P43 and signal peptide SP_hapR_. Therein, promoters Psrf, Pitu, Pfen resulted in about 100 U/mL activity of the protease BSP-1 (Jiang et al., 2022). In addition, two promoters of PhaII and PykzA with high expression levels in *Bacillus* were also selected (Guan et al., 2016; Rao et al., 2020). Firstly, we successfully constructed the expression vectors pHY-PhaII-SP_hapR_-*hapR*, pHY-Pitu-SP_hapR_-*hapR*, pHY-Psrf-SP_hapR_-*hapR*, pHY-Pfen-SP_hapR_-*hapR*, and pHY-PykzA-SP_hapR_-*hapR*. Subsequently, the five plasmids were transformed into *B. amyloliquefaciens* HZ12 to obtain corresponding recombinant strains HZ/pHY-PhaII-SP_hapR_-*hapR*, HZ/pHY-Pitu-SP_hapR_-*hapR*, HZ/pHY-Psrf-SP_hapR_-*hapR*, HZ/pHY-Pfen-SP_hapR_-*hapR*, and HZ/pHY-PykzA-SP_hapR_-*hapR* were obtained.

After fermentation of the recombinant strains for 48 h, the protease activities of the six strains were determined, and the results were compared in Figure 7A. Obviously, the activity of the P43-mediated strain was significantly higher than that of the other five promoters. Furthermore, we analyzed the proteins in the supernatant by SDS-PAGE. As shown in Figure 7B, all six promoter-regulated protease HapR were successfully expressed in the recombinant strain. Under the P43 promoter, the protease HapR (about 27 KDa) exhibited a much thicker band than that of other promoters, which was consistent with the results of the enzyme activity assay. Above data indicated that the strong constitutive promoter P43 was more suitable for the expression of the protease HapR. In the previous study, Jiang et al. also revealed that the protease BSP-1 achieves the maximum enzyme activity under the regulation of promoter P43 (Jiang et al., 2022). In another study, the promoter PHpaII-mediated aminopeptidase (AP) activity was higher than that of P43 (Guan et al., 2016). The reason may be due to the fact that different promoter is suitable for different target gene.

### 3.7 Effects of different signal peptides on the expression levels of protease HapR

**FIGURE 8:**
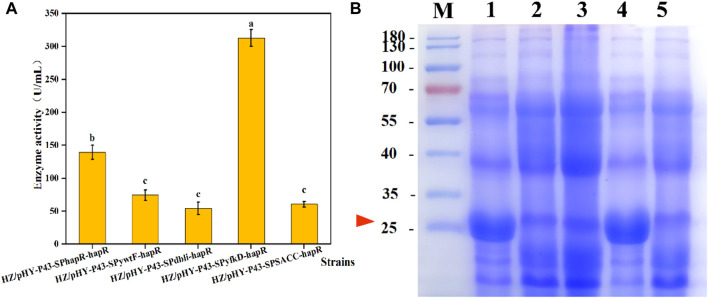
Effects of different signal peptides on expression levels of protease HapR. **(A)** Comparison of extracellular protease activities of recombinant strains with different signal peptides. **(B)** SDS-PAGE analysis of the fermentation supernatant of different strains. M: Marker; 1–5: HZ/pHY-P43-SP_hapR_-*hapR*, HZ/pHY-P43-SP_SACC_-*hapR*, HZ/Phy-P43-SP_ywtF_-*hapR*, HZ/pHY-p43-SP_yfkD_-*hapR*, and HZ/pHY-p43-SP_dbli_-*hapR*.

The selection of an appropriate signal peptide is one of the important strategies to enhance the extracellular expression of a target protein (Hemmerich et al., 2016). Many studies have shown that signal peptide optimization can enhance the production of recombinant proteins, but there is no specific approach to predict the ideal signal peptide for a given known protein (Mori et al., 2015; Hemmerich et al., 2016; Zhao et al., 2024). In order to screen a signal peptide matching the protease HapR, four promising signal peptides derived from *Bacillus* were compared with the native signal peptide of HapR. We successively constructed recombinant strains HZ/pHY-P43-SP_SACC_-*hapR*, HZ/pHY-P43-SP_ywtF_-*hapR*, HZ/pHY-P43-SP_yfkD_-*hapR*, and HZ/pHY-P43-SP_dbli_-*hapR*, which were compared with the HZ/pHY-P43-SP_hapR_-*hapR*. The five recombinant strains were fermented for 48 h, and the protease activity was shown in Figure 8A. The results showed that the recombinant strain HZ/pHY-P43-SP_yfkD_-*hapR* with signal peptide SP_yfkD_ had the highest protease activity, reaching up to 312.75 U/mL, which was 124% higher than that of the recombinant strain HZ/pHY-P43-SP_hapR_-*hapR*. Meanwhile, all of these strains showed distinct bands around 27 kDa by SDS-PAGE analysis, and the protease HapR was successfully expressed in the recombinant bacteria (Figure 8B). Especially, the target band of HZ/pHY-P43-SP_yfkD_-*hapR* was the thickest one. Notably, the signaling peptides SP_SACC_, SP_ywtF_, and SP_dbli_ exhibited weaker expression levels compared to SP_HapR_. The results demonstrated that obtaining a signal peptide suitable for protease HapR secretion was an effective strategy to increase its expression level.

### 3.8 Effects of different host strains on the expression activity of protease HapR

**FIGURE 9:**
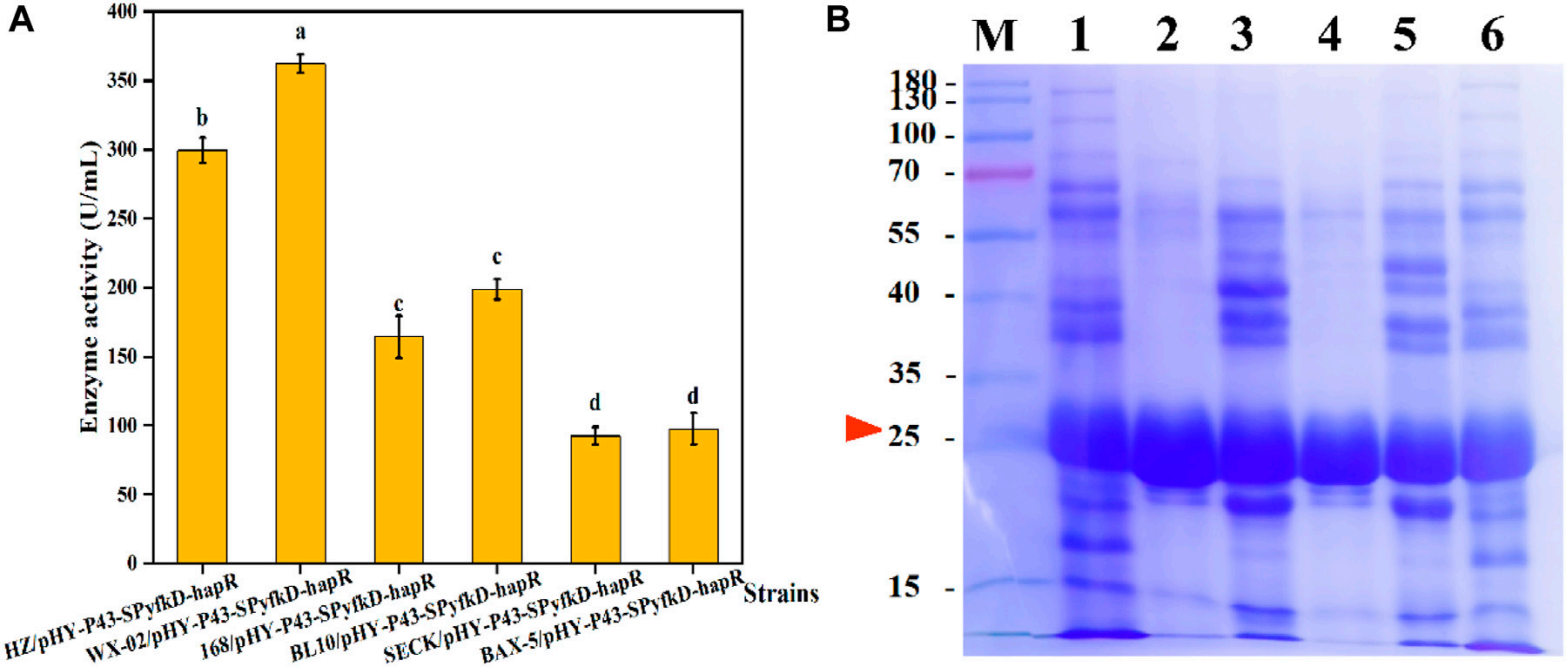
Effects of different host strains on expression levels of protease HapR. **(A)** Comparison of extracellular protease activities of recombinant strains with different host strains. **(B)** SDS-PAGE analysis of the fermentation supernatant of different strains. M: Marker; 1–6: HZ/pHY-P43-SP_yfkD_-*hapR*, WX-02/pHY-P43-SP_yfkD_-*hapR*, 168/pHY-P43-SP_yfkD_-*hapR*, BL10/pHY-P43-SP_yfkD_-*hapR*, SECK/pHY-P43-SP_yfkD_-*hapR*, and BAX-5/pHY-P43-SP_yfkD_-*hapR*.

Based on the above studies, we screened the promoters and signal peptides suitable for protease HapR expression. It was found that signal peptides caused different enzyme expression levels in different host strains (Hemmerich et al., 2016). For specific signal peptide-target protein combinations, the host strains context also affects the secretion efficiency (Freudl, 2018). The *Bacillus* species serve as the safe and important microbial host strains for the industrial production of enzymes, and they can be used for the secretion and production of extracellular proteases (Qian et al., 2023). Therefore, we selected five representative *Bacillus* expression systems for host strain screening. The plasmid pHY-P43-PyfkD-*hapR* was transformed into *B. licheniformis* WX-02, *B. subtilis* 168, *B. licheniformis* BL10, *B. subtilis* SECK, and *B. amyloliquefaciens* BAX-5, respectively. After 48 h of fermentation of the six recombinant strains, the protease activity of each recombinant strain was determined. As shown in Figure 9A, recombinant strain WX-02/pHY-P43-SP_yfkD_-*hapR* showed the highest protease activity of 384.27 U/mL, which was 167% higher than that of recombinant strain HZ/pHY-P43-SP_hapR_-*hapR*. Moreover, SDS-PAGE analysis revealed that the HapR protease was successfully expressed in all the six host strains (Figure 9B). Furthermore, the target band of the recombinant strain WX-02/pHY-P43-SP_yfkD_-*hapR* was thicker than that of the other five recombinant strains. SDS-PAGE analysis and enzyme activity assay experiments showed that the host strain WX-02 was more suitable for the expression of the protease HapR, regulated by the signal peptide SP_yfkD_. This study constructed an efficient recombinant strain for protease expression, which had the potential for the industrial production of protease for degradation of tobacco proteins.

## 4 Conclusion

This study screened an efficient protease-producing strain *B*. *velezensis* WH-7. Then, 103 known and putative proteases were predicted from *B*. *velezensis* WH-7 by whole genome sequencing analysis and annotation. Subsequently, 7 main proteases genes were mined, including five serine proteases genes (*hapR*, *ispA*, *vpr*, *epr*, *htrA*) and two metalloproteases (*ydcK*, *nprE*). By heterologous expression in *B. amyloliquefaciens* HZ-12, the gene *hapR* was demonstrated to encode the most efficient protease HapR. Moreover, the specific catalysis mechanism of HapR was further explained through amino acid sequence alignment analysis. Furthermore, the protease activity of HapR was enhanced by 167% after optimizing expression components such as promoter, signal peptide and host bacteria. To sum up, this study obtained an efficient protease-producing strain and corresponding gene resources, which would promote the development of enzyme preparations for degradation of tobacco proteins.

## Data Availability

The original contributions presented in the study are publicly available. The genome sequence data can be found here: https://www.ncbi.nlm.nih.gov/nuccore/CP145817. The 16S rRNA gene sequence data can be found here: https://www.ncbi.nlm.nih.gov/nuccore/PP380005.
