# Supplementary material for: Genetic identification and expression optimization of a novel protease HapR from Bacillus velezensis
Source: Front Bioeng Biotechnol. 2024 Mar 13;12:1383083. doi: 10.3389/fbioe.2024.1383083 (PMC10966715; doi:10.3389/fbioe.2024.1383083)
Supplement: Supplementary file 1 [file Table1.DOCX]

Supporting information

**Genetic identification and expression optimization of a novel protease HapR from *Bacillus velezensis***

Zhenying Han^1^, Changwen Ye^1,2*^, Xinyu Dong^1^, Chenchen Chen^1^, Dian Zou^1^, Kuo Huang^1,2^, Xuetuan Wei^1,3,4*^

^1^State Key Laboratory of Agricultural Microbiology, College of Food Science and Technology, Huazhong Agricultural University, Wuhan 430070, China

^2^Zhengzhou Tobacco Research Institute of China National Tobacco Corporation, Zhengzhou 450001, China

^3^Shenzhen Institute of Nutrition and Health, Huazhong Agricultural University, Wuhan, 430070, China.

^4^Shenzhen Branch, Guangdong Laboratory for Lingnan Modern Agriculture, Genome Analysis Laboratory of the Ministry of Agriculture, Agricultural Genomics Institute at Shenzhen, Chinese Academy of Agricultural Sciences, Shenzhen, 518000, China.

**Corresponding author*.

*E-mail address: weixuetuan@mail.hzau.edu.cn (Xuetuan Wei);* *yectsrc@163.com (Changwen Ye)*

*Postal address*: No. 1 Shizishan Street, Hongshan District, Wuhan 430070, Hubei, P. R. China

**Table S1 Sampling information for strain screening used in this study.**

| **Ecological Zone** | **Provinces** |
| --- | --- |
| Southwest Plateau Ecological Area | Yunnan, Sichuan |
| Guizhou Guangxi Mountain Ecological Area | Guizhou, Guangxi |
| Wuling Qinba Ecological Area | Hubei, Chongqing, and Shaanxi |
| Huanghuai Plain Ecological Area | Henan |
| The Nanling Mountain Hilly Ecological Area | Hunan, Jiangxi, Anhui, and Guangdong |
| Wuyi Hilly Ecological Area | Fujian |
| Yimeng Hilly Ecological Area | Shandong |
| Northeast China Plain Ecological Area | Heilongjiang, Jilin, and Liaoning |

**Table S2 Strains and plasmids used in this study.**

| **Strains/** **plasmid** | **Characteristics** | **Source** |
| --- | --- | --- |
| Strains |  |  |
| *E. coli* DH5α | supE44 ΔlacU169 (f 80 lacZΔM15) hsd R17 recA1 gyrA96 thi1 relA1 | Stored in lab |
| *B. licheniformis* WX-02 | Wide-type host strain  (CCTCC M208065) | Stored in lab |
| *B. subtilis* 168 | The strain with P43 promoter | Stored in lab |
| *B. amyloliquefaciens* HZ-12 | Wide-type | Stored in lab |
| *B. licheniformis* BL10 | *B. licheniformis* WX-02 deficient in *hag*,*mpr*,*vpr*,*aprX*,*epr*,*bpr*,*wprA*,*aprE*,*amyL*,  *bprA* | Stored in lab |
| *B. amyloliquefaciens* BAX-5 | *B. amyloliquefaciens* HZ-12 deficient in *epr*, *nprE*, *aprE*, *aprX*, *mpr* | Stored in lab |
| *B. subtilis* SECK | Wide-type | Stored in lab |
| *B. velezensis* WH-7 | Wide-type | This study |
| HZ-12/pHY-*ydcK* | HZ-12 with pHY-*ydcK* | This study |
| HZ-12/pHY-htrA | HZ-12 with pHY-*htrA* | This study |
| HZ-12/pHY-ispA | HZ-12 with pHY-*ispA* | This study |
| HZ-12/pHY-*vpr* | HZ-12 with pHY-*vpr* | This study |
| HZ-12/pHY-*epr* | HZ-12 with pHY-*epr* | This study |
| HZ-12/pHY-*hapR* | HZ-12 with pHY-*hapR* | This study |
| HZ-12/pHY-*nprE* | HZ-12 with pHY-*nprE* | This study |
| HZ-12/pHY300 | HZ-12 with pHY300 | This study |
| HZ-12/pHY-PhaII-SP_hapR_-*hapR* | HZ-12 with pHY-PhaII-SP_hapR_-*hapR* | This study |
| HZ-12/pHY-P43-SP_hapR_-*hapR* | HZ-12 with pHY-P43-SP_hapR_-*hapR* | This study |
| HZ-12/pHY-Pitu-SP_hapR_-*hapR* | HZ-12 with pHY-Pitu-SP_hapR_-*hapR* | This study |
| HZ-12/pHY-Psrf-SP_hapR_-*hapR* | HZ-12 with pHY-Psrf-SP_hapR_-*hapR* | This study |
| HZ-12/pHY-Pfen-SP_hapR_-*hapR* | HZ-12 with pHY-Pfen-SP_hapR_-*hapR* | This study |
| HZ-12/pHY-PykzA-SP_hapR_-*hapR* | HZ-12 with pHY-PykzA-SP_hapR_-*hapR* | This study |
| HZ12/pHY-P43-SP_hapR_-*hapR* | HZ-12 with pHY-P43-SP_hapR_-*hapR* | This study |
| HZ-12/pHY-P43-SP_SACC_-*hapR* | HZ-12 with pHY-P43-SP_SACC_-*hapR* | This study |
| HZ-12/pHY-P43-SP_ywtF_-hapR | HZ-12 with pHY-P43-SP_ywtF_-hapR | This study |
| HZ-12/pHY-P43-SP_yfkD_-*hapR* | HZ-12 with pHY-P43-SP_yfkD_-*hapR* | This study |
| HZ-12/pHY-P43-SP_dbli_-*hapR* | HZ-12 with pHY-P43-SP_dbli_-*hapR* | This study |
| WX-02/pHY-P43- SP_yfkD_ -*hapR* | HZ-12 with pHY-P43-SP_yfkD_-*hapR* | This study |
| 168/pHY-P43- SP_yfkD_ -*hapR* | 168 with pHY-P43-SP_yfkD_-*hapR* | This study |
| BL10/pHY-P43- SP_yfkD_ -*hapR* | BL10 with pHY-P43-SP_yfkD_-*hapR* | This study |
| SECK/pHY-P43- SP_yfkD_ -*hapR* | SECK with pHY-P43-SP_yfkD_-*hapR* | This study |
| BAX-5/pHY-P43- SP_yfkD_ -*hapR* | BAX-5 with pHY-P43-SP_yfkD_-*hapR* | This study |
| pHY300PLK | *E. coli*−*Bacillus* shuttle vector for gene expression, Apr, Tet | This study |
| pHY- *hapR* | pHY300PLK+P43+*hapR*+Tamyl | This study |
| pHY- *ydcK* | pHY300PLK+P43+*ydcK*+Tamyl | This study |
| pHY- *ispA* | pHY300PLK+P43+*ispA*+Tamyl | This study |
| pHY- *vpr* | pHY300PLK+P43+*vpr*+Tamyl | This study |
| pHY- *nprE* | pHY300PLK+P43+*nprE*+Tamyl | This study |
| pHY- *htrA* | pHY300PLK+P43+*htrA*+Tamyl | This study |
| pHY- *epr* | pHY300PLK+P43+*epr*+Tamyl | This study |
| pHY-PhaII-SP_hapR_-*hapR* | pHY300PLK+PhaII+SP_hapR_ + *hapR* + Tamyl | This study |
| pHY-Pitu-SP_hapR_-*hapR* | pHY300PLK+Pitu+SP_hapR_+ *hapR* + Tamyl | This study |
| pHY-Psrf-SP_hapR_-*hapR* | pHY300PLK+Psrf +SP_hapR_+*hapR* + Tamyl | This study |
| pHY-Pfen-SP_hapR_-*hapR* | pHY300PLK+Pfen+SP_hapR_+*hapR* + Tamyl | This study |
| pHY-PykzA-SP_hapR_-*hapR* | pHY300PLK+PykzA+SP_hapR_+ *hapR*+Tamyl | This study |
| pHY-P43-SP_hapR_-*hapR* | pHY300PLK+P43+SP_hapR_+*hapR* +Tamyl | This study |
| pHY-P43-SP_SACC_-*hapR* | pHY300PLK+P43+SP_SACC_+*hapR* + Tamyl | This study |
| pHY-P43-SP_ywtF_-*hapR* | pHY300PLK+P43+SP_ywtF_+*hapR* + Tamyl | This study |
| pHY-P43-SP_yfkD_-*hapR* | pHY300PLK+P43+SP_yfkD_+*hapR* + Tamyl | This study |
| pHY-P43-SP_dbli_-*hapR* | pHY300PLK+P43+SP_dbli_+*hapR* + Tamyl | This study |

**Table S3 Primers used in this study.**

| Primer name | Sequence of primer (5’to 3’) |
| --- | --- |
| P43-F | CGGAATTCTGATAGGTGGTATGTTTTCG |
| P43-R | CGGGATCCGTGTACATTCCTCTCTTACCTATAATG |
| Psrf-F | GCTTTGCCCAAGCTTGACAAAAATGTCATGAAAGAATCGT |
| Psrf-R | GCCTCTCATTCTAGAGGGTAAAAAGTTATTTCCATATTGTCATAC |
| Pitu-F | GCTTTGCCCAAGCTTTAATTTCTGACACAATAATGCCAATAGC |
| Pitu-R | GCCTCTCATTCTAGAGAGATTCCTCCGATCATATTGAAC |
| PhaII-F | GCTTTGCCCAAGCTTGGTGGAGATTTTTTGAGTGATCT |
| PhaII-R | GCCTCTCATTCTAGATAAATCGCTCCTTTTTAGGTGG |
| Pfen-F | GCTTTGCCCAAGCTTCAAAAATGGGCGGAATTTTTCAC |
| Pfen-R | GCCTCTCATTCTAGAAATGGCAGTTTTATCCTCCAGC |
| PykzA-F | GCTTTGCCCAAGCTTGAAATATTGATGTGACACTTGAAGTTG |
| PykzA-R | GCCTCTCATTCTAGAATTTGTATCTAGTTGTTATATTTCCCTTTCT |
| SP_SACC_-F | GAATGTACACTCTAGAATGAAAAAGAGACTGATTCAAGTCA |
| SP_SACC_-R | GTTTGATTTCCCTGCTGCATCTGCCGAAAATGC |
| SP_ywtF_-F | GAATGTACACTCTAGAAtggaagaacgatctcaacg |
| SP_ywtF_-R | GTTTGATTTCCCTGCtgcgtaggtgccgac |
| SP_yfkD_-F | GAATGTACACTCTAGAATGATGAAAAAGCTATTTCATTCCAC |
| SP_yfkD_-R | GTTTGATTTCCCTGCTTTCGCGTGGATGGGC |
| SP_SACC_-F | GAATGTACACTCTAGAATGAAAAAGAGACTGATTCAAGTCA |
| SP_SACC_-R | GTTTGATTTCCCTGCTGCATCTGCCGAAAATGC |
| TamyL-F | GCAGCTGCACAATAAAAGAGCAGAGAGGACGGATT |
| TamyL-R | GCTCTAGACGCAATAATGCCGTCGCACT |
| *ydcK*-F | GCTCTAGAATGTATGCGAACGGTGAATT |
| *ydcK*-R | CGGGATCCTTAAAAAATTCTTTTGATTTTTCC |
| *htrA*-F | GCTCTAGAATGATGGATAACTATCGTGATGAA |
| *htrA*-R | CGGGATCCTTAAGAAGATGTTTCTTCTTTTTGA |
| *ispA*-F | GCTCTAGAATGTATCCACTCGCAAGCAT |
| *ispA*-R | CGGGATCCTCAGAAAGACAGCAGCTGTG |
| *vpr*-F | GCTCTAGAATGAAAAAAGGAATCATCCGT |
| *vpr*-R | CGGGATCCTTATTCAATGATAAAAGGTTTTTCA |
| *epr*-F | GCTCTAGAATGGCCGCGGCCT |
| *epr*-R | CGGGATCCTTATTTCACCCGAATCGCA |
| *hapR*-F | GCTCTAGAATGAGAGGCAAAAAGGTATGG |
| *hapR*-R | CGGGATCCTTACTGAGCTGCCGCCTG |
| *nprE*-F | GCTCTAGAATGAGTAATCACAATTGGACGC |
| *nprE*-R | CGGGATCCTTATGATCTCACTTGGGCGG |
| pHY300-F | GTTTATTATCCATACCCTTAC |
| pHY300-R | CAGATTTCGTGATGCTTGTC |
